# Performance-based approach for movement artifact removal from electroencephalographic data recorded during locomotion

**DOI:** 10.1371/journal.pone.0197153

**Published:** 2018-05-16

**Authors:** Evyatar Arad, Ronny P. Bartsch, Jan W. Kantelhardt, Meir Plotnik

**Affiliations:** 1 Center of Advanced Technologies in Rehabilitation, Sheba Medical Center, Tel Hashomer, Ramat Gan, Israel; 2 Department of Physics, Bar-Ilan University, Ramat Gan, Israel; 3 Institute of Physics, Martin-Luther-University, Halle (Saale), Germany; 4 Department of Physiology and Pharmacology, Sackler Faculty of Medicine, Tel-Aviv University, Tel Aviv, Israel; 5 Sagol School of Neuroscience, Tel Aviv University, Tel Aviv, Israel; Fondazione Santa Lucia Istituto di Ricovero e Cura a Carattere Scientifico, ITALY

## Abstract

The appreciation for the need to record electroencephalographic (EEG) signals from humans while walking has been steadily growing in recent years, particularly in relation to understanding gait disturbances. Movement artefacts (MA) in EEG signals originate from mechanical forces applied to the scalp electrodes, inducing small electrode movements relative to the scalp which, in turn, cause the recorded voltage to change irrespectively of cortical activity. These mechanical forces, and thus MA, may have various sources (e.g., ground reaction forces, head movements, etc.) that are inherent to daily activities, notably walking. In this paper we introduce a systematic, integrated methodology for removing MA from EEG signals recorded during treadmill (TM) and over-ground (OG) walking, as well as quantify the prevalence of MA in different locomotion settings. In our experiments, participants performed walking trials at various speeds both OG and on a TM while wearing a 32-channel EEG cap and a 3-axis accelerometer, placed on the forehead. Data preprocessing included separating the EEG signals into statistically independent additive components using independent component analysis (ICA). We observed an increase in electro-physiological signals (e.g., neck EMG activations for stabilizing the head during heel-strikes) as the walking speed increased. These artefact independent-components (ICs), while not originating from electrode movement, still exhibit a similar spectral pattern to the MA ICs–a peak at the stepping frequency. MA was identified and quantified in each component using a novel method that utilizes the participant’s stepping frequency, derived from a forehead-mounted accelerometer. We then benchmarked the EEG data by applying newly established metrics to quantify the success of our method in cleaning the data. The results indicate that our approach can be successfully applied to EEG data recorded during TM and OG walking, and is offered as a unified methodology for MA removal from EEG collected during gait trials.

## Introduction

Surface electroencephalography (EEG) allows humanity a glimpse of our minds through the electrical output generated by the vast networks of neurons in our brains. The faint electrical signals arise from the joint activity of countless neurons, recorded using the EEG electrodes. Due to the delicate nature of the recorded signal it is easily overshadowed by various artefacts such as eye blinks, muscles activations, electromagnetic noise and movement artefacts[1,2]. The latter practically constraining the modern EEG to be recorded only during stationary settings.

EEG signals recorded during gait activity reflect neural mechanisms associated with healthy or impaired leg movements[3]. In the past decade, EEG signals were recorded during treadmill (TM) walking [4–7], identifying a systematic modulation of EEG spectral amplitude during the gait cycle and coupling of EEG recordings and electromyography recorded from the lower limbs. Some of the studies involving EEG recordings during human locomotion addressed gait disturbances, for example, in persons with Parkinson's disease and in particular the debilitating phenomenon of freezing of gait[8–12]. Due to the dynamic nature of the aforementioned experiments, much effort is involved in studying and removing movement artefacts [13–15], with some studies concluding that more sophisticated tools are needed to properly clean gait-related artifacts [2]. It also became apparent that data preprocessing is an important first step for MA removal because of the inherent complexity of the EEG data. However, currently there are no EEG MA studies that utilize an advanced preprocessing tool (i.e., PREP pipeline [16]) for improving the decomposition algorithm’s (i.e., Independent Component Analysis (ICA)) performance. The field of ICA algorithms has also evolved, and today better decomposition algorithms are available [17]. The objective of this study was to remove EEG MA by developing a new framework that combines the most advanced algorithms in preprocessing and signal decomposition analysis with our own novel methodology for MA identification, and to test the results using recently published EEG benchmarking metrics [15].

Movement artefacts (MA) in EEG signals originate from mechanical forces applied on the scalp electrodes, inducing small electrode movements relative to the scalp which, in turn, cause the recorded voltage to change regardless of cortical activity. It was previously claimed that movement artifact should be removed in order to study electro-cortical activity during locomotion[13]. However, according to recent studies, EEG data recorded during walking is likely to contain substantial MA that cannot be removed using traditional signal processing methods[2].

Various methods have been proposed for the removal of MA from EEG signals. Gwin et al., [13] first removed an MA template from the stride-epoched data using a 20-stride moving average, and then applied independent component analysis (ICA), a source-separation algorithm, to further clean the data. Leutheuser et al., [17] later compared two ICA algorithms, the common InfoMax as well as AMICA (Adaptive-Mixture ICA), in terms of their performance in reduction of EEG artefacts, and found that the AMICA algorithm outperformed the InfoMax. Later, Onikura et al. [14] suggested an ICA based method to remove head-movement MA by high pass filtering components whose temporal correlation coefficient with a head accelerometer crossed a predefined threshold. Kline et al. [2] tested the use of stride-locked moving average subtraction and Daubechies wavelet transform to remove walking MA of various speeds. Automatic subspace reconstruction (ASR) [18] is another novel method for MA removal in which infected segments of the data are processed using baseline data and principal component analysis (PCA). While not all of the above methods were reported to successfully remove MA, they unanimously noted that caution should be exercised as not to remove neural data along with the MA, leading to subject specific thresholds, manual inspection, etc. In addition, they all utilized different benchmarking metrics for performance evaluation, an issue that was thoroughly addressed by Oliveira et al. [15] who described metrics for benchmarking EEG technologies during whole body motion. In this paper we primarily introduce an integrated, novel methodology for removing MA from EEG signals recorded during treadmill (TM) and over-ground walking, as well as inspect the EEG signal for the prevalence of such artefact in different locomotion settings. We tested our proposed approach using state of the art benchmarking metrics [15] and equipment. The methodology, that incorporates different parts of past studies while adding novelty of its own, was aimed at finding a fine line between retaining as much neural data as possible and reducing MA.

## Methods

### Participants

The study included 5 young, healthy adult (mean age ± *SD*: 30.5 ± 5.31) participants. All participants gave their written informed consent prior to entering the study. The experimental protocol was approved by the ethics institutional review board for experiments involving human participants in the Chaim Sheba Medical Center.

### Data collection; signal measurement, apparatus

The participants were fitted with a 32-electrode wireless EEG system (eego sports 32 pro by ANT-Neuro, The Netherlands), which utilizes passive-wet electrodes arranged in the 10–20 system. Impedances were kept under 20 kOhm (cross-subject mean: 7.19 ± 3.77 *K*Ω), while channels with impedance of over 20 kOhm were excluded from the analysis. Using adhesive tape, an accelerometer (eego sports 32 pro by ANT-Neuro, The Netherlands) was placed on the midline of the participants’ forehead in order to determine the mechanical forces that were applied on the EEG electrodes during locomotion. An instrumented dual belt TM equipped with bi-lateral force plates was used (R-Mill, ForceLink, The Netherlands. EEG, accelerometer and ground reaction forces (GRF) data were recorded simultaneously during walking at 1024, 1024 and 120 Hz respectively. In addition, all trials were video-recorded.

### Experimental protocol

**Fig 1:**
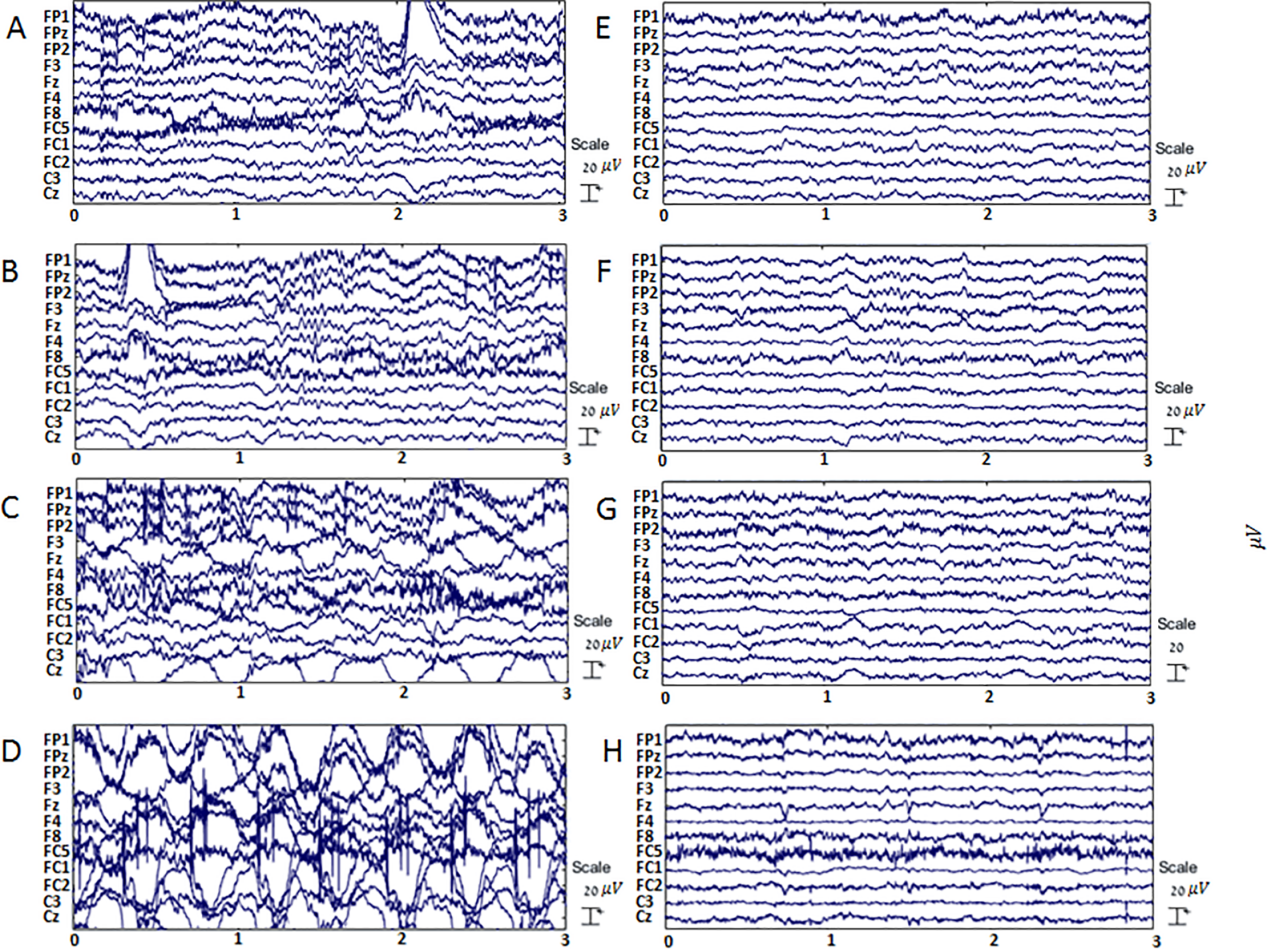
Exemplary EEG data recorded during different walking speeds before and after removal of movement artifacts. Panels A,B,C and D depict high pass filtered EEG data recorded during TM walking at speeds of 0.4, 0.8, 1.6 and 2.2m/s, respectively. To the right, panels E, F, G and H depict the same time segments in the data after movement artefact removal. The x axis represents time in seconds, the y axis shows the voltage vs. time for 10 electrodes (voltage curves are shifted vertically for clarity).

The experiment began with the recording of a one-minute sitting-baseline (BL), followed by 1-minute during which the participants were asked to nod their head back and forth in a comfortable frequency. The participants then performed six walking trials, each lasting two minutes. Firstly, four trials were performed on a TM at increasing speeds (0.4, 0.8, 1.6 and 2.2 m/s) (Fig 1), these were followed by two over-ground (OG) trials, first at the participant’s natural pace and another later with elevated walking speed. OG trials were performed in a 24 m long corridor and walking speeds for these trials were determined using the average walking time for completing a predefined 10 m segment of the corridor. Each participant’s OG walking speeds were derived from video recordings and accelerometer data.

### Data preprocessing

EEG Data preprocessing and analysis was performed in MATLAB [The MathWorks Inc., Natick, MA] using EEGLAB [19], fitted with the PREP extension [16] and custom scripts as follows:

1. As MA may interfere with Channel rejection (CR), the latter was performed using PREP according to BL data only and then applied to the participant's full, continuous, dataset. Criterions for CR were (Parametric thresholds for the PREP GUI appear in parentheses):
  1 Standard deviation (5)
  2 High frequency noise (5).
  3 Correlation with neighboring channels (0.4).
2. The channel-rejected, continuous, dataset was then processed by:
  1 Data de-trending by high-pass filtering; 1 Hz cutoff frequency.
  2 Line noise removal at 50Hz and its harmonics using CleanLine.
  3 Re-referencing of the signals to an average reference.
3. The continuous EEG data was cropped to trial specific datasets and each dataset was process by:
  1 Running the AMICA algorithm [20]
  2 Removing EOG, EMG and other non-movement artefact component by visual inspection and comparing the components’ spatial distribution, time course and spectrograms to typical artefact patterns as outlined in [1].
  3 Removal of artefacts is performed using EEGlab’s graphical user interface.

### Independent component analysis, MA component identification and removal from the EEG data

**Fig 2:**
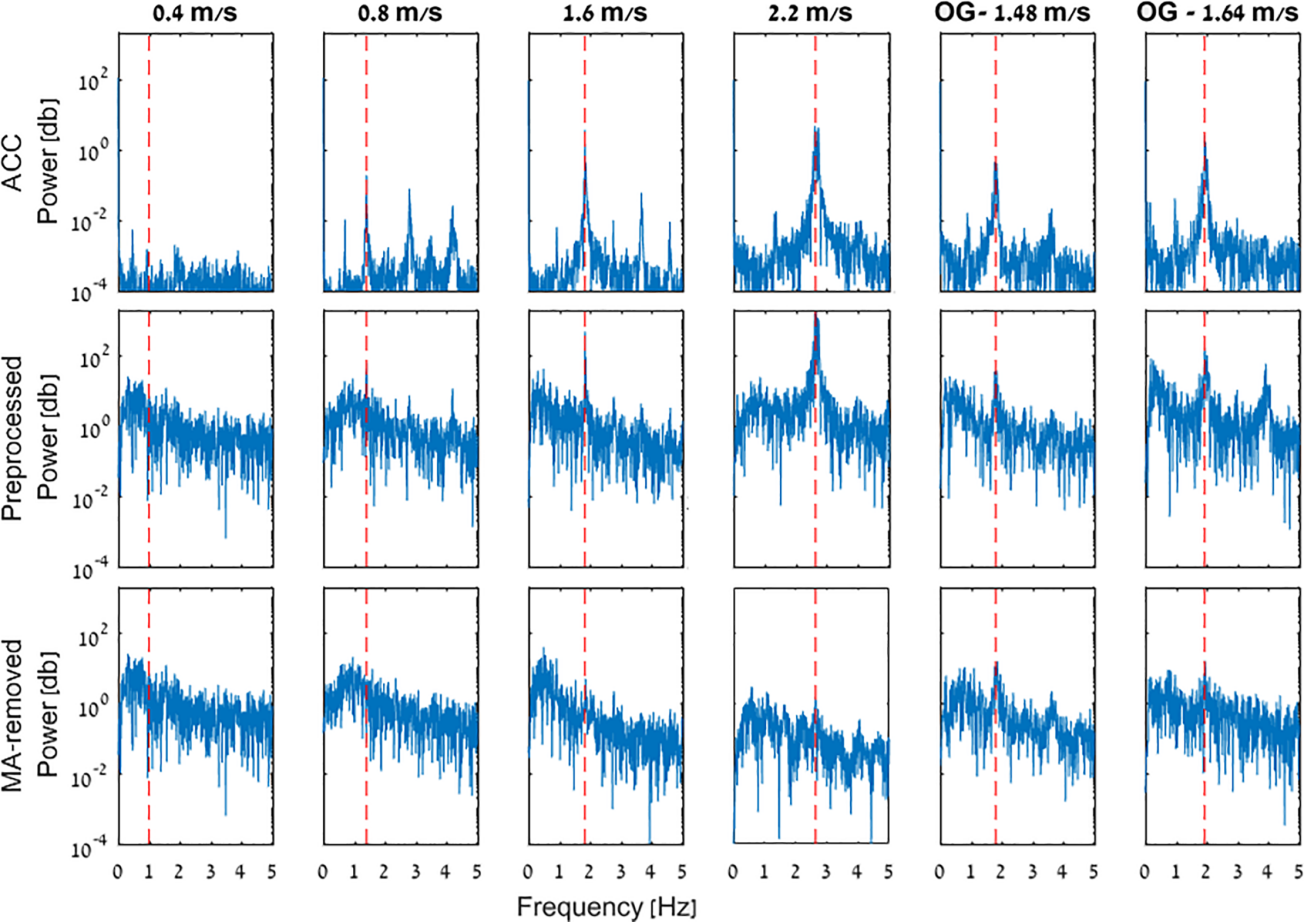
Cz electrode and accelerometer power spectra comparison. The top row depicts the accelerometer’s power spectra in the various walking trials, while the middle and bottom rows depict the power spectra of the Cz electrode after preprocessing and MA-removal, respectively. X and Y axes of the plots stand for frequency and spectral power, respectively.

All remaining components and vertical accelerometer data were transformed to the spectral domain using Fast Fourier transform (FFT). The average stepping frequency (ASF) of each trial was derived from the power spectrum of the accelerometer’s vertical component using peak detection (Fig 2) and verified against the recorded video (i.e., counting steps in a time unit) and heel strike (HS) detection using the GRF data.

The MA components have a unique spectral signature compared to their neural counterparts—a tall peak at the stepping frequency surrounded by relatively low amplitude. Since EEG data components usually have most of their spectral energy in the lower frequencies, a median is used to verify that indeed the MA independent components (ICs) spectral signature is present.

To assess the amount of MA, each IC was given an MA prevalence (MAP) score, calculated as

$$MAP_{Component}=\frac{Power\;at\;ASF}{Median(0-5\;Hz\;spectral\;power)}$$

Where ‘Power at ASF’ denotes the component’s spectral peak at the average stepping frequency, and 'Low frequency power median' is the power spectral density’s median in the 0–5 Hz band. The MAP score is calculated as the ratio between a component’s power at the stepping frequency and the median of the spectral power in the 0–5 Hz band. Since components marked as MA are removed entirely we wanted to make sure little to no neural data was removed along with them. After inspecting many MA and non-MA ICs’ frequency spectra, we came to the conclusion that a ratio of 80 can serve as a classifying threshold aiming to remove components containing mostly MA while retaining as much neural data as possible. A MAP score of 80 means that there is 80 times more spectral power in the average stepping frequency compared to its surroundings in the 0–5 Hz range (i.e., where most of the spectral power resides in non-MA components).

**Fig 3:**
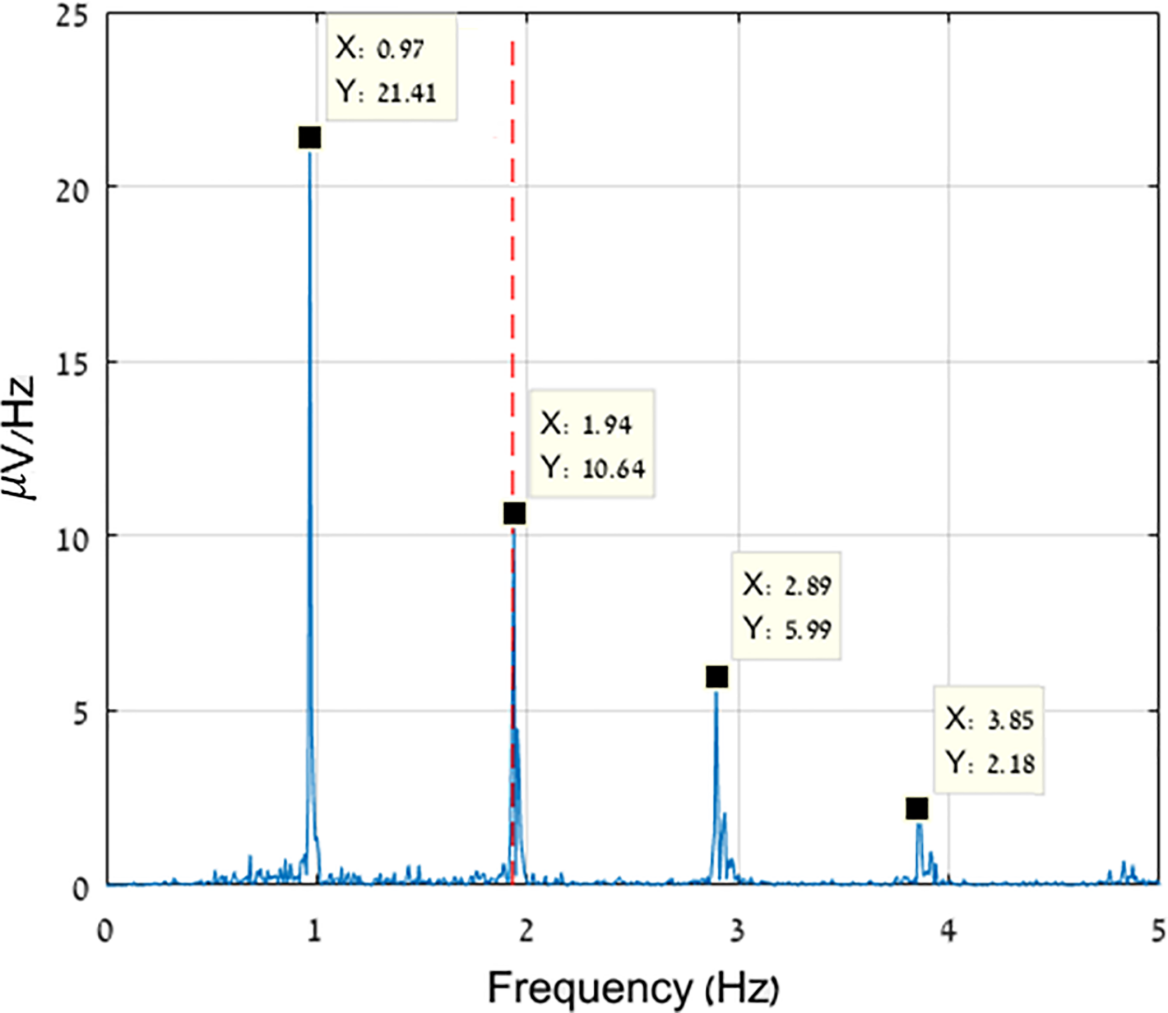
Decaying harmonics at 0.5 multiples of the stepping frequency. Frequency spectrum of an exemplary AMICA component, decomposed from a 1.6 m/s TM trial. Each spectral peak’s frequency and amplitude are depicted in the data-tips. The stepping frequency, as derived from the accelerometer, is shown as a red dashed line.

Additionally, we reviewed the power spectra of the different components and discovered another pattern of MA component spectra. This pattern featured decaying sub-harmonics at 0.5 multiples of the stepping frequency (Fig 3) and is related to lateral sway while walking, in line with previous studies[21]. These MA components were removed as well.

### Validation

In order to determine the prevalence of MA in the cleaned data and to avoid over-cleaning, the EEG data were benchmarked using two metrics. We utilized a metric described by Oliveira et al. called the walking/sitting (W/S) ratio [15]. The W/S ratio is calculated by dividing the spectral power in the 5–80 Hz band, containing the theta (5–8 Hz), alpha (9–13 Hz), beta (13–30 Hz) and gamma (30–80 Hz) brain oscillations, of a walking trial by the spectral power in the same band of the sitting BL. The W/S Ratio determines changes in EEG spectral content related to movement where a ratio that is larger than 1 suggests the existence of MA in the EEG data and a W/S ratio smaller than 1 may indicate the EEG data were over-cleaned. We note, however, that although the W/S ratios should ideally be 1, this would require the same continuous electro-cortical activity between seated and walking conditions. Previous studies indicated a power drop in the alpha (9–13 Hz) and beta (13–30 Hz) brain oscillations during motor activity when compared to a resting baseline [22], as well as a related drop in W/S ratios during locomotion in a different study [15]. These explain why W/S ratios that are only slightly below 1 are not necessarily an indicator of over-cleaning. We utilized the previously described drop in alpha and beta power to assess the physiological validity of our results by calculating the W/S ratio specifically for the alpha and beta bands during the different walking trials, before and after the proposed MA-removal methodology was applied.

To compare our method to the current state-of-the-art in MA removal, we also cleaned the data using ASR and compared the results using the previously described benchmarking criteria. ASR applies Principal Component Analysis (PCA) to the EEG data in a moving window, decomposing it into subspaces which are compared to a clean BL segment. The subspaces that are identified as noisy are reconstructed using an un-mixing matrix which was derived from the BL using PCA. By so, ASR automatically eliminates eye-blinks, muscle and movement artefacts. We referred to data prior to MA removal, data who’s MA was cleaned using our proposed method and data pruned using ASR as ‘Preprocessed’, ‘AMICA’ and ‘ASR’, respectively. The results of these comparisons were statistically analyzed using a two-way ANOVA by ranks (Related-samples Friedman’s test) for comparing the different methods and walking speeds.

#### Statistical analysis

Statistical analysis was performed with the IBM SPSS statistics 21 software. Significance level for all test was set to α = 0.05. In order to assess the effect of the MA induced by the increasing speeds as well as their effect on the EEG data, we analyzed the parameters obtained from preprocessed-only, pruned using AMICA and pruned using ASR datasets.

We evaluated each methods’ ability to remove MA from EEG data recorded during increasing walking speeds, using the W/S ratios, as well as alpha and beta bands’ spectral power parameters. This was performed using a two-way ANOVA by ranks (Related-samples Friedman’s test) for comparing the different walking speeds within each method.

Later, to assess the difference in performance between our proposed method and the current state-of-the-art (i.e., ASR), we performed a Related-samples Wilcoxon signed rank test, directly comparing the two methods, whereas the walking speeds now serving as the within variables. This was performed utilizing the 3 parameters described in the previous chapter.

## Results

The faster a person walks—the more GRFs he/she will be subjected to (Fig 1, Plotnik et al, 2013 [23]) and hence more forces will be applied on the scalp electrodes. Thus, generally speaking, the faster one’s walking speed—the more MA-infected the recorded EEG data will be, as demonstrated in Fig 1. As can also be seen in Fig 1, this is particularly true for the Cz electrode, that is the most prone to MA due to its location at the top of the scalp [2].

### Cz electrode and accelerometer spectral comparison

We begin by comparing the frequency spectra of the Cz electrode and of the accelerometer’s vertical component in Fig 2. Presented are data from all 6 walking trials (TM and OG) of a typical participant (i.e., HU971), where the peak in the accelerometer’s power spectrum corresponds to the participant’s stepping frequency. It can be seen that (a) the spectral peak of the accelerometer matches to the spectral peak in the Cz electrode (e.g., Fig 2, 2.2 m/s) (b) the spectral peak’s amplitude in the EEG and accelerometer data increased along with the walking speed in the trials before MA removal (c) MA removal considerably reduced the aforementioned peak.

### W/S ratios at various speeds and conditions

**Fig 4:**
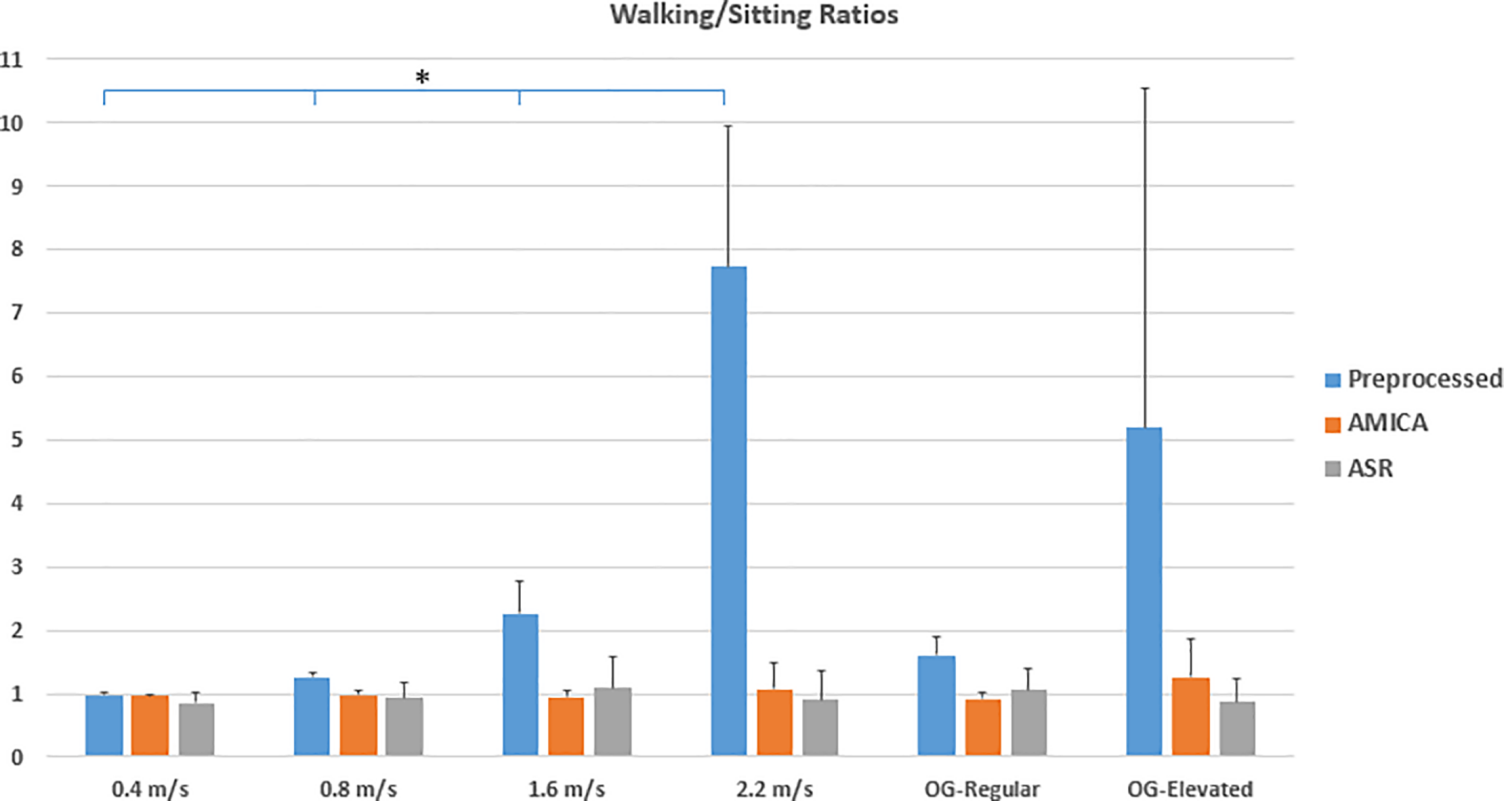
Walking/Sitting (W/S) ratios. W/S ratios are presented for the various walking trials for the preprocessed-only, AMICA-cleaned and ASR-cleaned EEG data in blue, orange and gray, respectively. X and y axes depict the various trials and the corresponding W/S ratios, respectively. The mean cross-subject regular and elevated OG walking speeds were 1.37 and 1.81 m/s, respectively. Shown are the cross-subject group average and standard deviation. For the preprocessed data (blue), all multiple comparisons are significantly different for TM walking (p<0.01, as indicated by the asterisks), as opposed to AMICA and ASR cleaned data.

As displayed in Fig 4, the W/S ratio for the trials prior to MA removal increased dramatically in conjunction with the walking speeds, while trials cleaned by our method and ASR displayed a ratio ranging between 0.87 and 1.25 across all trials While the data cleaned by AMICA and ASR are not affected by speed (*χ*^2^(5) = 1.2,*p* = 0.94; *χ*^2^(5) = 5.6,*p* = 0.34, respectively), the preprocessed-only data was clearly speed-dependent (*χ*^2^(5) = 22.94,*p* < 0.0001). For pairwise comparisons see Fig 4. Ratios >1 were encountered in the AMICA 2.2 m/s and OG-elevated trials (1.07 and 1.25, respectively) as well as the ASR 1.6 m/s and OG-regular trials (1.09 and 1.05, respectively). The W/S ratios rendered by ASR presented higher SD values when compared to our method, with the exception of OG-elevated. Note that although the MA manifests predominantly in the stepping frequency (i.e., below 5 Hz), it is clearly apparent in the 5–80 Hz band. Directly comparing our proposed method and ASR produced no statistically significant difference (*Z* = 6,*p* = 0.68).

### MA distribution across the scalp

**Fig 5:**
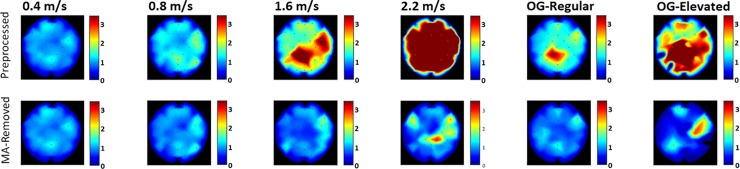
W/S ratios spatial distribution across the scalp. Quantitative comparison between 6 EEG signals before and after MA removal. The spectral energy in the 5–80 Hz band was compared to the measured baseline’s spectral energy of the same frequency band. The values, spatially distributed across the scalp, depict the spectral energy ratios between walking and sitting (i.e., baseline), around the stepping frequencies associated with the different speeds (warmer colors reflect higher ratios). The mean cross-subject regular and elevated OG walking speeds were 1.37 and 1.81 m/s, respectively. Black dots depict scalp electrodes.

The scalp distribution maps portrays a more detailed picture of Fig 4 W/S ratios by spatially displaying the various electrodes’ ratios as they are spatially spread across the scalp. Cross-subject scalp distribution maps of the W/S ratios across the 32 electrodes and in all trials are presented in Fig 5. Each electrode is represented by a dot while inter-electrode values were generated using spline interpolation. It can be seen how in the TM setting, the lowest speed (i.e., 0.4 m/s) presents practically no MA to begin with while right after, at 0.8 m/s, MA begin to appear and increase along walking speeds where in 2.2 m/s the W/S ratios values are well over 3 and the scalp distribution maps are saturated. Further, as a control, we applied the proposed methodology to artificial ‘simulated walking noise’ added to a baseline recording, and confirmed that after applying our method, W/S ratios dropped to 1.002 (see full description of this procedure in S3 File).

### Alpha and beta bands analysis during motor activity

**Fig 6:**
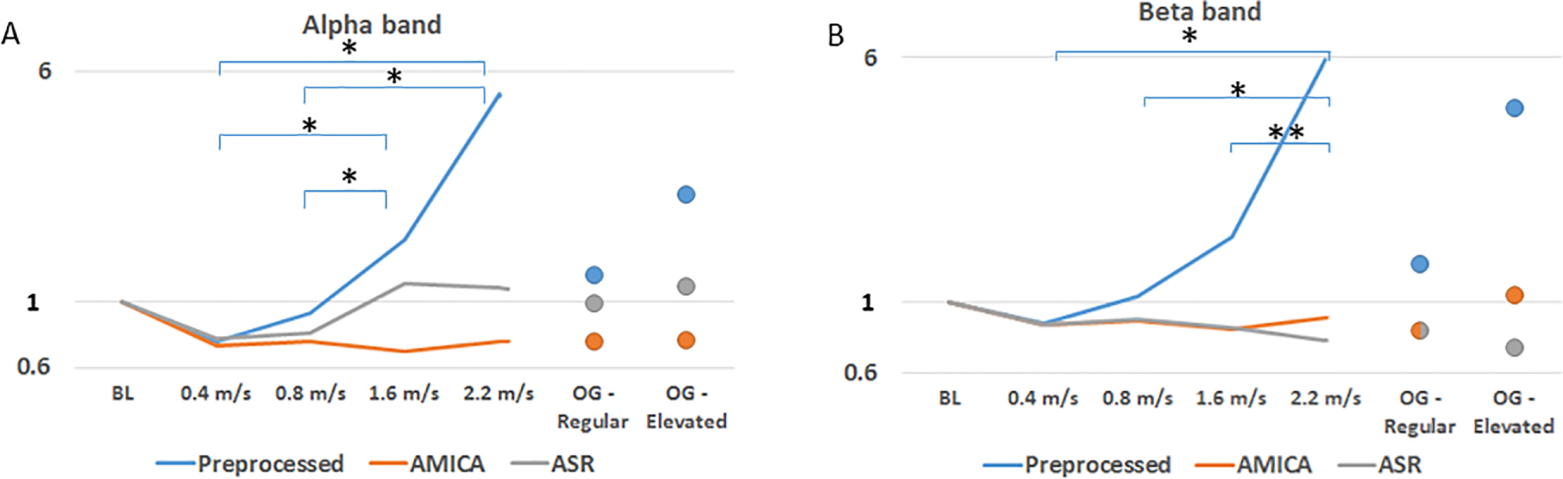
Alpha and beta bands analysis during motor activity. W/S ratios specifically calculated for the alpha [7.5–12 Hz] and beta [13–30 Hz] bands are presented in panels A and B, respectively. Ratios of the preprocessed-only, AMICA and ASR cleaned data are displayed in blue, orange and gray, respectively. The mean cross-subject regular and elevated OG walking speeds were 1.37 and 1.81 m/s, respectively. X and y axes depict the various trials and band’s W/S ratio, respectively. For preprocessed data (blue), significance in the pairwise comparisons are indicated by asterisks (single asterisks denotes p<0.01 and double asterisks p<0.05). AMICA and ASR cleaned data showed no statistically significant power increase.

The energies of the alpha and beta bands were normalized in reference to each participant’s sitting baseline of the same band, in order to assess changes in alpha and beta power during locomotion. Fig 6 presents these values for each trial. It can be seen how prior to MA removal, the expected drop in energy is realized, for both bands only in the slowest speed, 0.4 m/s. Prior to MA removal, the data presented a power increase at higher speeds (1.6 m/s and 0.8 m/s and above for the alpha and beta bands, *χ*^2^(5) = 19.51,*p* = 0.002; *χ*^2^(5) = 17.8,*p* = 0.003, respectively). For pairwise comparisons see Fig 6.

Data cleaned by our proposed method consistently exhibited the expected power drop in both the alpha and beta bands across all trials, with the exception of OG-elevated. ASR, in comparison, presented the beta band’s power drop in most of the trials, but also displayed an unexpected increase in alpha power during the 1.6 m/s, 2.2 m/s and OG-elevated trials. Nevertheless, Friedman’s tests yielded non-significant results (*χ*^2^(5) = 1,*p* = 0.96 for our method and *χ*^2^(5) = 5.57,*p* = 0.35 for ASR), indicating that these deviations are non-significant. Directly comparing our proposed method and ASR in the alpha and beta bands, no statistically significant difference was found (*Z* = 7;*p* = 0.89 and *Z* = 7,*p* = 0.686).

### Increased electro-physiological signals during higher speeds

**Fig 7:**
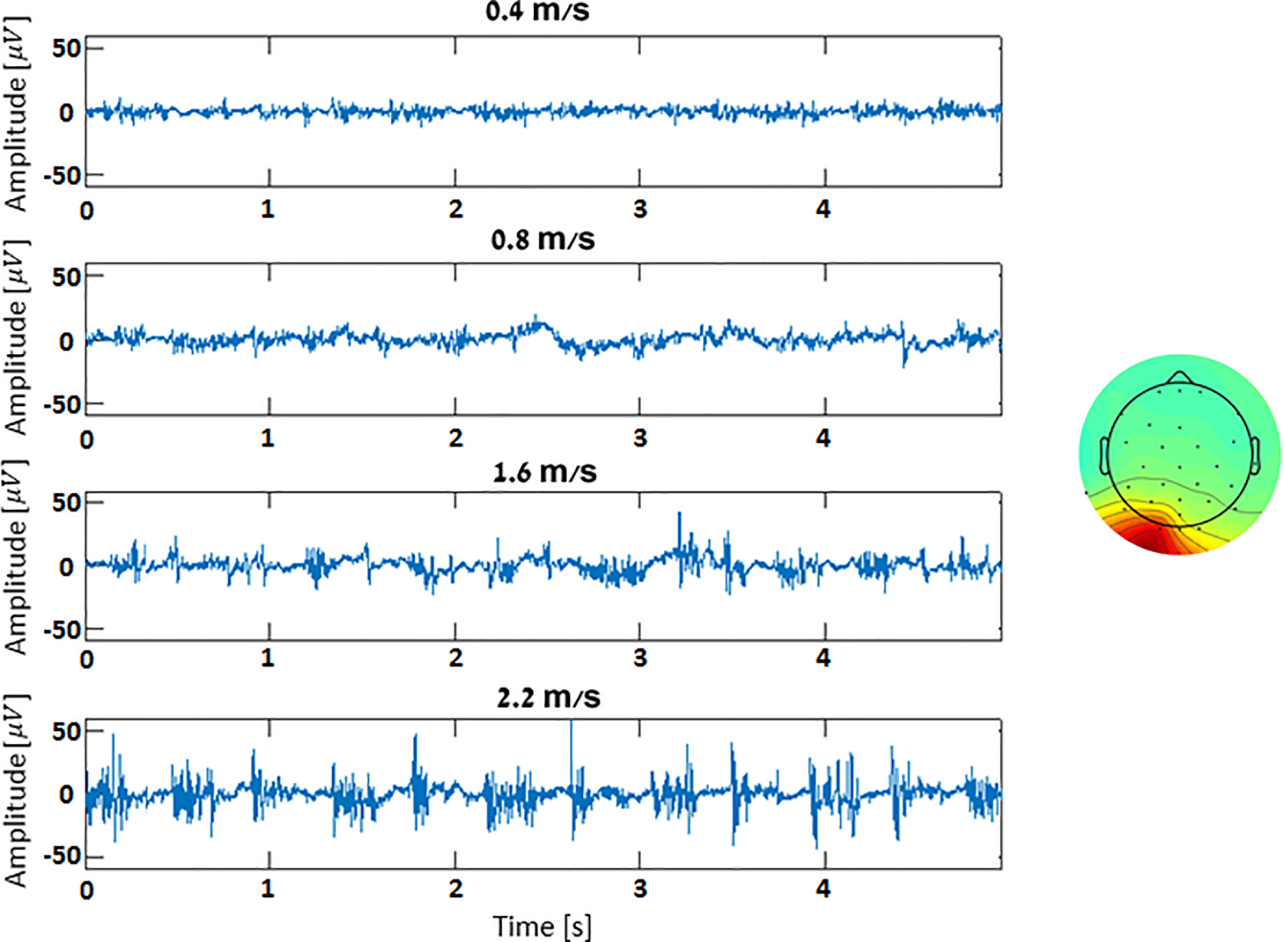
Increased electro-physiological signals during higher speeds. Presented are 10-second temporal segments of occipital neck muscle components, as separated by the AMICA algorithm (exemplary component map presented on the right), recorded during the various TM speeds of a single participant. Speeds increase from top to bottom, x and y axes depict time and amplitude [V], respectively.

Fig 7 depicts neck muscle activations during the various TM trials, as separated by the AMICA algorithm. An increase in component activation is apparent along with the walking speed (standard deviation (SD) values for the 0.4, 0.8, 1.6 and 2.2 m/s are 3.17, 3.5, 6.37 and 8.59, respectively).

## Discussion

In this article we have measured the effects of locomotion on recorded EEG data and proposed a novel methodology for the removal of the associated MA. Currently there are no EEG MA studies, that utilize an advanced integrated preprocessing tool (i.e., PREP pipeline), which greatly affects the decomposition algorithm’s (i.e., AMICA) performance. Our procedure was successfully applied for TM and OG walking alike, and is offered as a unified methodology for MA removal from EEG collected during gait trials.

In general, the proposed approach removes MA based on stepping frequency information. In order to substantiate this approach we began with presenting evidence pointing that there is, in fact, correlation between the frequency spectrum of the recorded, uncleaned, data from the EEG electrodes and the forces applied on the scalp during locomotion. Given the periodic nature of human locomotion, our analysis of the ICs’ frequency spectra serves as a straightforward and powerful way to detect and remove locomotion-originated MA. We also tested our methodology by applying it to data from baseline recordings with added artificial noise (i.e., simulating MA) and demonstrated that those artificial artefacts were successfully removed (see Figure A in S3 File).

Our approach offers a systematic way of evaluating MA through the analysis of spectral components. We propose a full ‘step by step’ approach from start to finish, as well as combine multiple state-of-the-art signal processing methods (i.e., PREP pipeline, AMICA) with our novel methodology for assessing and removing MA.

### Cz electrode and accelerometer spectral properties

Since the Cz electrode is the most prone to MA, we decided to examine this electrode’s power spectrum before and after MA removal to provide a worst-case scenario overview.

Looking at the vertical accelerometer data (Fig 2, top row), it is apparent that the faster the participant walked, the more MA was induced to the EEG data (i.e., wider, taller spectral peaks at the stepping frequency; Fig 2, middle row). This can be observed by the increasing amplitude of the spectral peaks along with the walking speed. Additionally, it can be seen that OG walking leads to more MA compared to TM at similar speeds.

### Differential considerations on MA removal for TM and OG walking

In the lowest speed, 0.4 m/s, MA was negligible (i.e., low MAP score) and therefore the power spectra of the preprocessed and MA-removed data are the same. The spectral peak in the stepping frequency grew as the TM’s speed was increased, while the higher speeds proved to be harder to clean (i.e., more EMG and MA components detected). Additionally, OG walking trials were harder to clean than trials performed on the TM because of two reasons: (i) Higher GRFs while walking OG, compared to similar TM speeds, due to the TM’s suspension dampening and (ii) The self-paced nature of OG walking results in small variations to the stepping frequency across the trial thus, providing a quasi-periodic signal that’s harder for the AMICA algorithm to separate from the neural data. On par with other observations in this study, the Cz electrode frequency spectra show our proposed method was able to prune the data from MA in all TM walking speeds and provide a substantial improvement in TM running and OG locomotion.

### W/S ratios at various speeds and conditions

The W/S ratio, as seen growing alongside the walking speed in Fig 4, offers an opportunity to examine the increase of spectral energy contents in the brainwaves band of EEG data recorded during locomotion. While at the lowest speed (i.e., 0.4 m/s), both pre and post MA removal ratios are close to 1, an increase at the W/S ratio is already apparent at 0.8 m/s, indicating the presence of MA in the EEG data. This increase further escalades intensely at higher speeds. The MA pruned data, on the other hand, presents ratios slightly below 1 for all walking speeds, both on the TM and OG, which is in line with previous studies [15] as well as studies that have demonstrated a reduction in alpha and beta power during motor activity [22].

It can also be observed that the W/S ratio’s standard deviation increased alongside the walking speed. These increasing SDs are a result of small differences in setup between subjects (i.e., small variations in the <20 impedance, different weight, walking style, sole cushioning, etc.). These differences, while miniscule at lower speeds, increase and hyper-manifest as the walking speed increases, and come to an extreme at OG locomotion. This points at how caution should be practiced when recording EEG during OG locomotion or TM running, due to the higher GRFs. We found no statistical difference in the performance at removing MA between our method and the current state-of-the-art (i.e., ASR). Nevertheless, our suggested method presents lower inter-electrode variance (i.e., smaller error bars in Fig 4), which suggests a more homogeneous removal of MA across the scalp and thus cleaner EEG data.

### MA distribution across the scalp

Looking at Fig 5, we see again how for the three TM-walking speeds, as well as the OG-regular, the proposed method is able to clean the data well while at the TM-running (i.e., 2.2 m/s) and OG-elevated trials some MA is still present in the signal. In the latter conditions, the MA constitutes a very large portion of the data. So much so, that the AMICA algorithm struggles to separate it from the neural data, resulting in mixed neural-MA components. This gives us an idea about the method’s abilities and limitations in various walking conditions. Additionally, It can be seen how MA appears first (i.e., at lower speeds) mainly at the top of the scalp, in the area around the Cz electrode, and later spreads to the peripheral areas, consistent with prior studies[2].

### Alpha and beta bands analysis during motor activity

Past studies have demonstrated a link between motor activity and a reduction in the alpha and beta bands’ power of the recorded EEG [22,24]. Moreover, a more prominent drop has been shown in the alpha band when compared to the beta band. This property can serve as a good physiological validity benchmark of the EEG data, both prior to and after the MA-removal process, as depicted in Fig 6. Looking at the preprocessed-only data, an increase is evident in both the alpha and Beta bands’ normalized spectral power. ASR displayed better values compared to the preprocessed datasets, but on the other hand also presented an unexpected increase in power evident in the alpha band during TM trials at speeds of 1.6 m/s and above, as well as in the OG-elevated trial, which may originate from non-cortical artifacts.

Due to the slighter change in the beta band’s power during motor activity, its power drop is more susceptible to MA as it diminishes in lower speeds then the drop in the alpha band’s power (the power drop in the preprocessed data is diminished at 1.6 and 0.4 m/s for the alpha and beta bands, respectively). This delicate nature of the beta band also explains why it is first, and only, at displaying an increase in the MA-removed data (i.e., at the OG-elevated trial, which also displays the highest ratio after MA-removal).

The alpha and beta bands analysis shows how MA removal is important in order to reveal physiological changes in EEG that may be hidden beneath the MA. Moreover, our results demonstrate that MA removal is essential in order to detect physiological changes in EEG during locomotion.

### Increased electro-physiological signals during higher speeds

An interesting observation we made using AMICA is how electro-physiological signals, such as the neck muscles activations as separated by the algorithm, increased with the walking speed. We presume these increasing activations are in order to stabilize the participant’s head during heel strikes at the growing TM speeds. While these components do not originate from scalp electrodes movement, and thus will probably be categorized as EMG artefacts and removed at the preprocessing stage, they exhibit a power spectrum similar to the MA’s–a peak in the stepping frequency. This serves as an example of how physiological systems (e.g., muscles, heart rate, etc.) are engaged more intensively when walking faster–therefore possibly introducing electro-physiological (i.e., non-MA) noise to the recorded EEG.

Thus, while our bodies engage physiological systems (e.g., muscles, heart rate, etc.) more intensively to walk faster, further care is needed to isolate the noise related to the elevated activity within the EEG signal and remove it, in order to obtain a signal that reflects primarily brain activity.

## Supporting information

S1 File Data for Fig 4. (XLSX)

S2 File Data for Fig 6. (XLSX)

S3 File Supplementary material. (DOCX)

## References

[1] Chaumon M, Bishop DVM, Busch NA. A practical guide to the selection of independent components of the electroencephalogram for artifact correction. J Neurosci Methods. 2015;250: 47–63. doi: 10.1016/j.jneumeth.2015.02.025 25791012 10.1016/j.jneumeth.2015.02.025

[2] Kline JE, Huang HJ, Snyder KL, Ferris DP. Isolating gait-related movement artifacts in electroencephalography during human walking. J Neural Eng. 2015;12: 046022 doi: 10.1088/1741-2560/12/4/046022 26083595 10.1088/1741-2560/12/4/046022 PMC4946867

[3] Peterson DS, Horak FB. Neural Control of Walking in People with Parkinsonism. Physiology. 2016;31: 95–107. doi: 10.1152/physiol.00034.2015 26889015 10.1152/physiol.00034.2015 PMC4888974

[4] Gwin JT, Gramann K, Makeig S, Ferris DP. Electrocortical activity is coupled to gait cycle phase during treadmill walking. NeuroImage. 2011;54: 1289–1296. doi: 10.1016/j.neuroimage.2010.08.066 20832484 10.1016/j.neuroimage.2010.08.066

[5] Petersen TH, Willerslev-Olsen M, Conway BA, Nielsen JB. The motor cortex drives the muscles during walking in human subjects: Cortico-muscular coupling during walking. J Physiol. 2012;590: 2443–2452. doi: 10.1113/jphysiol.2012.227397 22393252 10.1113/jphysiol.2012.227397 PMC3424763

[6] Presacco A, Goodman R, Forrester L, Contreras-Vidal JL. Neural decoding of treadmill walking from noninvasive electroencephalographic signals. J Neurophysiol. 2011;106: 1875–1887. doi: 10.1152/jn.00104.2011 21768121 10.1152/jn.00104.2011 PMC3296428

[7] Haefeli J, Vögeli S, Michel J, Dietz V. Preparation and performance of obstacle steps: interaction between brain and spinal neuronal activity: Brain activity during obstacle stepping. Eur J Neurosci. 2011;33: 338–348. doi: 10.1111/j.1460-9568.2010.07494.x 21070395 10.1111/j.1460-9568.2010.07494.x

[8] Handojoseno AMA, Shine JM, Nguyen TN, Tran Y, Lewis SJG, Nguyen HT. The detection of Freezing of Gait in Parkinson’s disease patients using EEG signals based on Wavelet decomposition. Conf Proc Annu Int Conf IEEE Eng Med Biol Soc IEEE Eng Med Biol Soc Annu Conf. 2012;2012: 69–72. doi: 10.1109/EMBC.2012.6345873 23365834 10.1109/EMBC.2012.6345873

[9] Handojoseno AMA, Shine JM, Nguyen TN, Tran Y, Lewis SJG, Nguyen HT. Using EEG spatial correlation, cross frequency energy, and wavelet coefficients for the prediction of Freezing of Gait in Parkinson’s Disease patients. Conf Proc Annu Int Conf IEEE Eng Med Biol Soc IEEE Eng Med Biol Soc Annu Conf. 2013;2013: 4263–4266. doi: 10.1109/EMBC.2013.6610487 24110674 10.1109/EMBC.2013.6610487

[10] Handojoseno AMA, Shine JM, Nguyen TN, Tran Y, Lewis SJG, Nguyen HT. Analysis and Prediction of the Freezing of Gait Using EEG Brain Dynamics. IEEE Trans Neural Syst Rehabil Eng. 2015;23: 887–896. doi: 10.1109/TNSRE.2014.2381254 25532186 10.1109/TNSRE.2014.2381254

[11] Shine JM, Handojoseno AMA, Nguyen TN, Tran Y, Naismith SL, Nguyen H, et al Abnormal patterns of theta frequency oscillations during the temporal evolution of freezing of gait in Parkinson’s disease. Clin Neurophysiol. 2014;125: 569–576. doi: 10.1016/j.clinph.2013.09.006 24099920 10.1016/j.clinph.2013.09.006

[12] Ly QT, Handojoseno AMA, Gilat M, Chai R, Martens KAE, Georgiades M, et al Detection of gait initiation Failure in Parkinson’s disease based on wavelet transform and Support Vector Machine. IEEE; 2017 pp. 3048–3051. doi: 10.1109/EMBC.2017.8037500 29060541 10.1109/EMBC.2017.8037500

[13] Gwin JT, Gramann K, Makeig S, Ferris DP. Removal of Movement Artifact From High-Density EEG Recorded During Walking and Running. J Neurophysiol. 2010;103: 3526–3534. doi: 10.1152/jn.00105.2010 20410364 10.1152/jn.00105.2010 PMC3774587

[14] Onikura K, Katayama Y, Iramina K. Evaluation of a Method of Removing Head Movement Artifact from EEG by Independent Component Analysis and Filtering. Adv Biomed Eng. 2015;4: 67–72. doi: 10.14326/abe.4.67

[15] Oliveira AS, Schlink BR, Hairston WD, König P, Ferris DP. Proposing Metrics for Benchmarking Novel EEG Technologies Towards Real-World Measurements. Front Hum Neurosci. 2016;10 doi: 10.3389/fnhum.2016.00188 27242467 10.3389/fnhum.2016.00188 PMC4861738

[16] Bigdely-Shamlo N, Mullen T, Kothe C, Su K-M, Robbins KA. The PREP pipeline: standardized preprocessing for large-scale EEG analysis. Front Neuroinformatics. 2015;9 doi: 10.3389/fninf.2015.00016 26150785 10.3389/fninf.2015.00016 PMC4471356

[17] Leutheuser H, Gabsteiger F, Hebenstreit F, Reis P, Lochmann M, Eskofier B. Comparison of the AMICA and the InfoMax algorithm for the reduction of electromyogenic artifacts in EEG data. IEEE; 2013 pp. 6804–6807. doi: 10.1109/EMBC.2013.6611119 24111306 10.1109/EMBC.2013.6611119

[18] Mullen TR, Kothe CAE, Chi YM, Ojeda A, Kerth T, Makeig S, et al Real-Time Neuroimaging and Cognitive Monitoring Using Wearable Dry EEG. IEEE Trans Biomed Eng. 2015;62: 2553–2567. doi: 10.1109/TBME.2015.2481482 26415149 10.1109/TBME.2015.2481482 PMC4710679

[19] Delorme A, Makeig S. EEGLAB: an open source toolbox for analysis of single-trial EEG dynamics including independent component analysis. J Neurosci Methods. 2004;134: 9–21. doi: 10.1016/j.jneumeth.2003.10.009 15102499 10.1016/j.jneumeth.2003.10.009

[20] Palmer JA, Kreutz-Delgado K, Makeig S. AMICA: An adaptive mixture of independent component analyzers with shared components. Swartz Cent Comput Neursoscience. 2012;

[21] Imai T, Moore ST, Raphan T, Cohen B. Interaction of the body, head, and eyes during walking and turning. Exp Brain Res. 2001;136: 1–18. doi: 10.1007/s002210000533 11204402 10.1007/s002210000533

[22] Tzagarakis C, West S, Pellizzer G. Brain oscillatory activity during motor preparation: effect of directional uncertainty on beta, but not alpha, frequency band. Front Neurosci. 2015;9 doi: 10.3389/fnins.2015.00246 26257597 10.3389/fnins.2015.00246 PMC4508519

[23] Plotnik M, Bartsch RP, Zeev A, Giladi N, Hausdorff JM. Effects of walking speed on asymmetry and bilateral coordination of gait. Gait Posture. 2013;38: 864–869. doi: 10.1016/j.gaitpost.2013.04.011 23680424 10.1016/j.gaitpost.2013.04.011 PMC4047486

[24] Kurz MJ, Proskovec AL, Gehringer JE, Becker KM, Arpin DJ, Heinrichs-Graham E, et al Developmental Trajectory of Beta Cortical Oscillatory Activity During a Knee Motor Task. Brain Topogr. 2016;29: 824–833. doi: 10.1007/s10548-016-0500-8 27277428 10.1007/s10548-016-0500-8 PMC5503148

