# Supplementary material for: Performance-based approach for movement artifact removal from electroencephalographic data recorded during locomotion
Source: PLoS One. 2018 May 16;13(5):e0197153. doi: 10.1371/journal.pone.0197153 (PMC5955591; doi:10.1371/journal.pone.0197153)
Supplement: S3 File — (DOCX) [file pone.0197153.s003.docx]

$$MAP_{Component}=\frac{Power at ASF}{Median(0-5 Hz spectral power)}$$

**Supplementary Material**

**Artificial MA induction and removal**

For this analysis, performed for verification, we used a BL recording and added noise to four neighboring electrodes (Cz, CP1, CP2 and Pz). To simulate MA, sine functions featuring multiples of the chosen simulated stepping frequency (i.e., 1.6 Hz) and decreasing amplitudes were used. The sine function translates to a pulse in the spectral domain, mimicking the MA components’ periodogram. Additionally, we added white Gaussian noise (WGN) to each electrode, featuring a signal-to-noise ratio of 10 dB.

Mathematically put, for each electrode:

$$EEG_{noisy\_A}=EEG_{BL}(t)+50\cdot\sin\left( 2\pi\cdot1.6\cdot t \right)+25\cdot\sin\left( 2\pi\cdot3.2\cdot t \right)+12\cdot\sin\left( 2\pi\cdot6.4\cdot t \right)$$

$$EEG_{noisy\_Final}=EEG_{noisy\_A}\left( t \right)+WGN(t)$$

The resulting EEG had a mean W/S ratio of 2.8143 over all EEG electrodes as well as a mean ratio of 14.1535 across the four noisy electrodes. The AMICA decomposition effectively separated the noise from the BL recording, resulting in the following component:


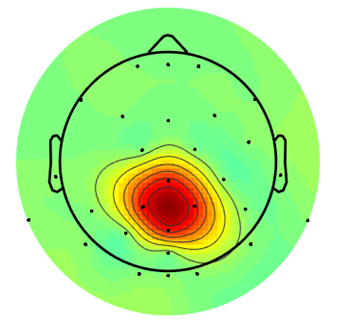


**Figure A. Component map spatially depicting the artificial MA, as decomposed by AMICA.** Black dots stand for EEG electrodes, green and red represent low and high amplitudes, respectively.

After removing the artificial MA component, the mean W/S ratio dropped to 1.002 over all EEG electrodes, as well as a mean ratio of 1.018 across the Cz, CP1, CP2 and Pz electrodes, nearing ideal decomposition of the noise and removal of the noise.
